# Understanding the dosing-time-dependent antihypertensive effect of valsartan and aspirin through mathematical modeling

**DOI:** 10.3389/fendo.2023.1110459

**Published:** 2023-03-08

**Authors:** Javiera Cortés-Ríos, Maria Rodriguez-Fernandez

**Affiliations:** Institute for Biological and Medical Engineering, Schools of Engineering, Medicine and Biological Sciences, Pontificia Universidad Católica de Chile, Santiago, Chile

**Keywords:** chronopharmacology, hypertension, mathematical modeling, blood pressure, circadian rhythm, valsartan, aspirin

## Abstract

Chronopharmacology of arterial hypertension impacts the long-term cardiovascular risk of hypertensive subjects. Therefore, clinical and computational studies have proposed optimizing antihypertensive medications’ dosing time (Ta). However, the causes and mechanisms underlying the Ta-dependency antihypertensive effect have not been elucidated. Here we propose using a Ta- dependent effect model to understand and predict the antihypertensive effect of valsartan and aspirin throughout the day in subjects with grade I or II essential hypertension. The model based on physiological regulation mechanisms includes a periodic function for each parameter that changes significantly after treatment. Circadian variations of parameters depending on the dosing time allowed the determination of regulation mechanisms dependent on the circadian rhythm that were most relevant for the action of each drug. In the case of valsartan, it is the regulation of vasodilation and systemic vascular resistance. In the case of aspirin, the antithrombotic effect generates changes in the sensitivity of systemic vascular resistance and heart rate to changes in physical activity. Dosing time-dependent models predict a more significant effect on systemic vascular resistance and blood pressure when administering valsartan or aspirin at bedtime. However, circadian dependence on the regulation mechanisms showed different sensitivity of their circadian parameters and shapes of functions, presenting different phase shifts and amplitude. Therefore, different mechanisms of action and pharmacokinetic properties of each drug can generate different profiles of Ta-dependence of antihypertensive effect and optimal dosing times.

## 1 Introduction

During the last years, the chronopharmacology of arterial hypertension has gained clinical relevance due to its impact on long-term cardiovascular risk (1–3). Chronopharmacological clinical trials have shown that antihypertensive medications from different pharmacological groups as angiotensin-converting enzyme (ACE) inhibitors, angiotensin II receptor blockers (ARBs), and calcium channel blockers (CCBs), have different antihypertensive effects when administered in the morning versus in the afternoon or at bedtime (4, 5). In addition, studies have shown that a low dose of aspirin, when administered at bedtime but not upon awakening, has an antihypertensive effect (6–8). However, understanding the regulatory mechanisms of blood pressure (BP) that drive a dosing-time-dependent effect has not been thoroughly explored.

The dosing-time-dependent effect of antihypertensive medications can be explained by circadian pharmacokinetic variations, which include changes in drug absorption, distribution, metabolism, and excretion processes, as well as circadian variation of therapeutic targets (pharmacodynamic changes) (9–11). On the one hand, pharmacokinetic changes are mainly produced by circadian changes in the rate of absorption, metabolism, and elimination of antihypertensive medications. Pharmacokinetic-pharmacodynamic models used to optimize the administration time of antihypertensive medications, showed that pharmacokinetic processes with lower constants (i.e., slower processes) are more dependent on dosing time than processes with higher constants (12). Therefore, the circadian dependence of the pharmacokinetic parameters of each antihypertensive medication may be different, even for those with the same mechanism of action. On the other hand, pharmacodynamic changes are due to circadian changes in physiological regulation mechanisms of BP, such as components of the renin-angiotensin-aldosterone system, norepinephrine, epinephrine, Na+ transporters, among others (13, 14), and changes in variables related to routine, such as physical activity, eating and sleep habits (15). These variables together result in a circadian BP pattern characterized by a morning rise, a postprandial deep, and a further fall during the night rest (a nighttime reduction of 10-20% compared to daytime BP is defined as a dipper pattern, and less than 10%, as a non-dipper pattern). Usually, pharmacodynamic models that describe the variation of BP during the day are composed of two-component periodic equations (12 and 24 hours) (16–18). But their parameters do not allow us to understand the mechanisms of the physiological regulation of BP. In order to understand and predict the circadian rhythm of BP, a model of four ordinary differential equations (ODEs) was recently published by our group (15). This ODEs model is capable of describing the variations of systemic vascular resistance (SVR), systolic blood pressure (SBP), heart rate (HR), and diastolic blood pressure (DBP) of dipper and non-dipper subjects with essential arterial hypertension, and receives physical activity (Act), norepinephrine (NE) and glycemia (glc) profiles as input. However, the time-dependent effect of antihypertensive medications was not included in this model.

Thus, circadian rhythm-dependent pharmacokinetic and pharmacodynamic variations may generate different BP profiles after treatment and variations in SVR and HR. Using previously published data on valsartan (an ARB) and aspirin (which is known for its anticoagulant effect at low doses) administered upon awakening versus at bedtime (6, 19), this work proposes the use of a dosing-time-dependent ODE model for the analysis of the time-dependent antihypertensive effect in patients with grade I or II essential hypertension. The model includes the circadian variation of parameters related to physiological variables that changed significantly after treatment and allowed to obtain effect predictions on BP, SVR, and HR dynamics through dosing times from 0 to 24 hours. Sensitivity analysis of circadian parameters showed a greater time-dependence of parameters related to vasodilation regulatory mechanisms in the case of valsartan and HR regulation in the case of aspirin. Finally, better antihypertensive results were obtained at bedtime administration for both valsartan and aspirin. For bedtime administration, valsartan was shown to be effective in lowering nocturnal BP and the morning surge of BP while lowering SVR, and aspirin was shown to reduce SVR and, to a lesser extent, BP and HR in the administration at bedtime but not upon awakening.

## 2 Materials and methods

### 2.1 Experimental data

Mean 24-h-dynamic data for physical activity, HR, SBP, and DBP were extracted using the Engauge Digitizer software from two independent single-dose studies of Hermida et al. (6, 19). Specifically, data from 148 non-dipper subjects with grade I or II hypertension before and after three months of valsartan treatment (160 mg/day) and data from 159 dipper subjects with grade I hypertension before and after three months of aspirin treatment (100 mg/day) at awakening versus bedtime administration. Notably, the time axis for all the variables was synchronized according to the 24 hours activity/rest cycle, so time 0 corresponds to the awakening time. Therefore, as reported by Hermida et al. (6, 19) for the valsartan and aspirin studies, the time 0 corresponds to the dosing time upon awakening and the time 15 to the dosing time for bedtime administration (15 hours after awakening). The extracted data were used to fit the previously published 4-ODEs model based on physiological BP regulation mechanisms, which describe changes in SVR, SBP, HR, and DBP over time (15). This 4-ODE model receives physical activity (Act), plasma norepinephrine (NE), and glycemia (glc) as inputs. Therefore, the same NE and glc data previously reported from the 4-ODE model were used to fit data from dipper (aspirin data) and non-dipper (valsartan data) subjects (15). As described in the work of Cortés-Ríos & Rodriguez-Fernandez, the systemic vascular resistance (SVR) data to fit the model was obtained using the following equation (20):


Eq. 1 $$SVR_{\left(dyn*\frac{s}{cm^{5}}\right)}\;=\frac{\left(\frac{2*SBP+DBP}{3}-CVP\right)*80}{SV*HR}$$

assuming an average stroke volume (*SV*) for subjects with essential hypertension of 79.5 ml (21) and replacing the SBP, HR and DBP extracted data. The mean right atrial pressure value for essential hypertension patients (5 mmHg) (22) was used instead of the central venous pressure (CVP) since, as previously reported, both values are similar given the low resistance of large vessels (23). The standard error of SVR was calculated from the standard error of the variables SBP, DBP, and HR.

### 2.2 Mathematical models

The previously published model that describes the variations of SVR $\left(dyn*\frac{s}{cm^{5}}\right),$ BP (*mmHg*) HR (*bpm*) and DBP (*mmHg*) over the integration time *(t)* is shown in equations 2-5 (15). Where Act (*counts*/*min*), NE (*ng*/*L*) and glc (*mmol*/*L*) are input variables (*v*_*input*_) and $k_{1}\;\left(dyn*\frac{s}{cm^{5}}\right)$ , *k*_*i*1_(*counts*/*min*), *n* (*unitless*), $k_{i2}\left(dyn*\frac{s}{cm^{5}}\right)$ $k_{3}\;\left(\frac{mmHg*cm^{5}}{dyn*s}\right)$ , *k*_*i*3_ (*bpm*), *k*_2_ (*bpm*), *k*_*i*4_ (*counts*/*min*), *k*_4_ (*mmHg* ^−1^), and *k*_5_ (*ng/L/*(*mmol/L*)^n^), are parameters (*p*). Parameters *k*_3_, *k*_4_ and *k*_5_ are equal to 1 and were incorporated into the model to ensure dimensional consistency. Therefore, the 4-ODEs model is determined by *t*,*v*_*input*_ and *p* (*Model*(*t*,*v*_*input*_,*p*)).


Eq. 2 $$\frac{dSVR}{dt}=k_{1}*\frac{k_{i1}}{k_{i1}+Act}*\frac{NE}{k_{5}*glc^{n}+NE}-\frac{SVR}{k_{i2}+SVR}*SVR$$

Eq. 3 $$\;\frac{dSBP}{dt}=k_{3}*SVR*\frac{HR}{k_{i3}+HR}-SBP$$

Eq. 4 $$\;\frac{dHR}{dt}=k_{2}*\frac{Act}{k_{i4}+Act}-k_{4}*DBP*HR$$

Eq. 5 $$\;\frac{dDBP}{dt}=k_{3}*SVR*\frac{HR}{k_{i3}+HR}-SBP$$

Then, the dosing-time-dependent effect of antihypertensives was included by defining a one-component periodic function to describe the variation of each of the parameters of equations 2-5 that change significantly at different dosing times (*Ta*) and do not have a high correlation (>0.95).


Eq. 6 $$p\left(Ta\right)=p_{before}+A\cos\left(W_{24}\left(Ta\right)+O\right)$$

Thus, the parameters after treatment of the Ta-dependent model depend on Ta (p(Ta)), and they are determined by the parameters: *p*_*before*_ (parameters before treatment), *A* (amplitude for 24 hours components), *W*_24_ (angular frequency, 2π/24) and *O* (acrophase for 24 hours component). It is important to note that *p*(*Ta*) does not depend on the integration time (*t*) of the system of differential equations (equations 2-5); the model parameters (*p*={*k*_1_, *k*_*i*1_, *n*, *k*_*i*2_, *k*_*i*3_, *k*_2_, *k*_*i*4_ }) defined by equation 6 depend on the dosing time (Ta) and the amplitude and acrophase estimated for the ones that changed significantly after treatment (*A*_*n*_, *A*_*k*_*i*1__,*A*_*k*_*i*2__,*A*_*k*_*i*3__,*A*_*k*_*i*4__,*A*_*k*_2__,*O*_*n*_,*O*_*k*_*i*1__,*O*_*k*_*i*2__,*O*_*k*_*i*3__,*O*_*k*_*i*4__,*O*_*k*_2__). Thus, the SVR, SBP, HR, and DBP variation over time (*t*) is defined by fixed *p* parameters for a specific Ta.

### 2.3 Parameter estimation

First, before and after treatment data for the awakening and bedtime administration groups were fitted independently using equations 2-5 for both valsartan and aspirin data. Subsequently, equation 6, which incorporates the variation of the parameters depending on the dosing time, was introduced for parameters that changed significantly in at least one of the two awakening or bedtime administration groups. Then, the parameters of equations 1-6 (time-dependent effect model) were fitted to the experimental data before and after treatment employing global optimization using a scatter search method (MEIGO) (24), the solver ode23s of MATLAB to integrate the model and the following objective function:


Eq. 7 $$F_{objetive}=\frac{1}{k*N}\sum_{i=1}^{k}\sum_{n=1}^{N}{\left(\frac{y_{\exp i}\left(t_{n}\right)-y_{\text{model }i\left(t_{n}\right)}}{\sigma_{i}\left(t_{n}\right)}\right)}^{2}$$

where N is the total number of mean experimental data per variable, *k* is the number of variables, *y_exp i_* is the mean experimental value of the *i* variable, *y_model i_* is the value predicted by the model and *σ*_*i*_ s the standard error from the data of the *i* variable.

### 2.4 Sensitivity and identifiability analysis

Sensitivity and identifiability analyses were performed for each model using the SENS_SYS third-party MATLAB function (25). SENS_SYS allowed obtaining the local sensitivity of each variable (SVR, SBP, HR, and DBP) for each parameter evaluated at the best parameter set. Sensitivity values for each experimental time (*t*_*n*_) were grouped in a sensitivity matrix (*S*_*ij*_(*t*_*n*_)), for i variables and j parameters. Then, according to the methodology described by Cortés-Ríos and Rodriguez-Fernandez (26), identifiability analysis was carried out using the Fisher information matrix (FIM) (equation 8), which was obtained from the local sensitivity matrix *(S*_*ij*_(*t*_*n*_)) and the covariance matrix (Q) (27). The covariance matrix was calculated using the standard error from the data.


Eq. 8 $$FIM=\sum_{n=1}^{N}S\left(t_{n}\right)\cdot Q\left(t_{n}\right)\cdot S{\left(t_{n}\right)}^{T}$$

The inverse of the FIM represents an approximation of the parameter estimation error covariance between the parameters j and h $(\sigma_{jh}^{2}={\left(FIM^{-1}\right)}_{jh)}$ and its diagonal is an approximation of the variance of the parameters (15, 27). Therefore, assuming a normal distribution, the 95% confidence interval (CI) of a parameter can be approximated by *p* ± 2*σ*_*jj*_ (28).


Eq. 9 $$\kappa_{jh}=\frac{\sigma_{jh}^{2}}{\sigma_{hh}\cdot \sigma_{jj}}$$

Thus, the correlation between parameters j and h (*κ*_*jh*_) can be calculated using equation 9 described by Ljung et al. (27).

### 2.5 T-test approximation

A two-tailed t-test approach was used to establish significant differences between before- and after-treatment independently estimated parameters (equations 2-5), for both the awakening and bedtime administration groups. Using independently estimated values of each parameter before (*p*_*before*_) and after treatment (*p*_*after*_), the approximation of the estimation error for each parameter (*σ*_*before* _ for *p*_*before*_ and *σ*_*after*_ for *p*_*after*_) and the number of subjects per treatment group (*n*_*group*_) the T value described by equation 10 was calculated for each treatment group (29).


Eq. 10 $$T=\frac{p_{before}-p_{after}}{\sqrt{\frac{\sigma_{before}^{2}}{n_{group}}+\frac{\sigma_{after}^{2}}{n_{group}}}\;}$$

For the awakening administration group of valsartan *n*_*group*_ is 72, and 76 for the bedtime administration group. In the case of aspirin, *n*_*group*_ is 77 for the awakening administration group and 82 for the bedtime administration group. Since the degrees of freedom (*df*) for all evaluated treatment groups is greater than (120 *df*=2*n*_*group*_−2), absolute T values greater than 1.96 were considered significant at 95% confidence level (p<0.05) and greater than 2.58 at 99% confidence level (p< 0.01) (29).

### 2.6 Circadian dependent-effect model simulations

Estimated parameters of the Ta-dependent model (*A* and *O* parameters of equation 6) allowed setting the Ta-dependent parameters (*p*(*Ta*)) and predicting the dynamics of SVR, SBP, HR, and DBP after valsartan and aspirin treatment across the full range of Ta (0 to 24 hours). Furthermore, estimated parameters using before-treatment data (*p*_*before*_) allowed describing the dynamics of the same variables in the absence of valsartan and aspirin. Therefore, the effect on the dynamics of SVR, SBP, HR, and DBP for each Ta was obtained by subtracting the dynamics predicted by the model using the parameters before treatment *Model*(*t*,*v*_*input*_, *p*_*before*_) and the dynamics predicted using the Ta-dependent parameters after treatment *Model*_*Ta*_(*t*,*v*_*input*_, *p*(*Ta*)) see equation 11.


Eq. 11 $$Effect\left(t,Ta,v_{input},p_{before},p(Ta)\right)=Model\left(t,v_{input},p_{before}\right)-Model_{Ta}\left(t,v_{input},p\left(Ta\right)\right)$$

Finally, effect units of each variable were expressed in the same corresponding units of each variable, i.e., dyn*s/cm^5^ for SVR, bpm for HR, and mmHg for SBP and DBP.

## 3 Results

### 3.1 Estimated parameters for awakening vs. bedtime administration

Valsartan and aspirin data before and after treatment were used to fit the non-Ta-dependent model independently. **Tables 1**, **2** show the estimated values and respective confidence intervals (CI, in the same units of each parameter) for each treatment group (before and after treatment for awakening and bedtime groups) for valsartan and aspirin, respectively. Since preliminary identifiability results showed a high correlation between parameters *k*_1_ and *k*_*i*2_, the parameter *k*_1_ was set to the value estimated using the before-treatment data to determine if there was a significant change in *k*_*i*2_ after treatment. Parameters that did not have significant changes after treatment are marked in gray in **Tables 1**, **2** (parameter *k*_1_ did not change because it was fixed in all groups). Parameters *k*_*i*1_, *n, k*_*i*2_, *k*_*i*3_, *k*_2_ and *k*_*i*4_ changed significantly after valsartan treatment both at awakening and at bedtime administration. Whereas only the *k*_*i*1_, *n, k*_*i*3_ and *k*_*i*4_ parameters changed in the administration upon awakening of aspirin, and only the parameters *n, k*_*i*2_, *k*_*i*3_, *k*_2_ and *k*_*i*4_ in the administration at bedtime of aspirin.

**Table 1:** Estimated parameters for awakening and bedtime administration of valsartan.

Parameter	Awakening group						Bedtime group					
	Before treatment		After treatment		T-value	p-value	Before treatment		After treatment		T-value	p-value
	Value	CI	Value	CI			Value	CI	Value	CI		
k1	601.60	293.96	601.6	278.86	0	p > 0.05	614.09	416.14	614.09	642.88	0	p > 0.05
n	2.10	0.52	2.41	0.34	8.47	p< 0.01	2.08	0.54	2.28	0.48	4.69	p< 0.01
ki2	4109.26	3019.06	3564.33	2713.96	2.28	p< 0.05	3923.80	4032.58	2564.19	4665.18	3.84	p< 0.01
ki1	631.15	196.24	567.69	178.88	4.06	p< 0.01	728.03	266.96	1979.16	1276.58	16.73	p< 0.01
ki3	770.99	18.96	767.57	20.26	2.09	p< 0.05	769.82	18.00	766.60	18.40	2.18	p< 0.05
ki4	16.36	1.32	17.53	1.48	9.94	p< 0.01	11.57	1.16	15.83	1.06	47.05	p< 0.01
k2	7274.70	114.12	6685.35	108.24	63.59	p< 0.01	6881.54	100.98	6528.63	91.18	45.23	p< 0.01

**Table 2:** Estimated parameters for awakening and bedtime administration of aspirin.

Parameter	Awakening group						Bedtime group					
	Before treatment		After treatment		T-value	p-value	Before treatment		After treatment		T-value	p-value
	Value	CI	Value	CI			Value	CI	Value	CI		
k1	491.40	204.96	491.40	312.42	0	p > 0.05	548.59	481.34	548.59	411.04	0	p > 0.05
n	2.80	0.36	2.66	0.42	4.42	p < 0.01	2.24	0.50	1.91	0.80	3.65	p < 0.01
ki2	4837.62	3539.68	4670.12	4877.80	0.49	p > 0.05	3150.04	4404.96	2591.03	3065.14	2.30	p < 0.05
ki1	1361.26	832.98	1038.90	576.18	5.59	p < 0.01	1486.44	858.46	1451.89	717.46	0.73	p > 0.05
ki3	764.94	16.16	756.36	15.98	6.63	p < 0.01	765.79	16.18	769.89	17.54	4.10	p < 0.01
ki4	21.61	1.32	17.28	1.02	45.64	p < 0.01	19.27	1.10	22.35	1.28	22.22	p < 0.01
k2	6961.67	93.78	6966.17	89.34	0.61	p > 0.05	7097.80	92.14	6805.75	96.06	56.16	p < 0.01

Estimated parameter values for valsartan before and after treatment showed that major differences exist between parameters related to SVR. Higher values of parameters *n* and *k*_*i*1_ show that SVR has a greater dependence on glycemia values and physical activity in the administration upon awakening, while a lower value of the parameter *k*_*i*2_ indicates a greater vasodilator effect when administered at bedtime (*k*_*i*2_ is related to the autoregulatory effect of vasodilation). The *k*_*i*3_ parameter related to the baroreflex regulation of BP increased both in the administration upon awakening and at bedtime, showing a slight improvement in baroreflex sensitivity. The parameters related to the regulation of HR, *k*_2_ and *k*_*i*4_ showed a greater dependence on physical activity (higher value of and a lower HR production constant (lower value of *k*_2_ both for administration upon awakening and at bedtime. However, changes in HR equation parameters did not generate changes in the HR profile after valsartan treatment, so they are probably due to adjustments for differences in baroreflex sensitivity (i.e., changes in parameters *k*_2_ and *k*_*i*4_ compensate changes in the negative component of equation 4).

In the case of aspirin, parameter *n* slightly decreased both for the administration upon awakening and at bedtime, showing a slight decrease in the sensitivity of SVR to changes in glycemia. While *k*_*i*2_ parameter decreased significantly only at bedtime; that is, there is only a significant effect on the regulation of vasodilation at bedtime administration. Furthermore, parameter *k*_*i*1_ only decreased significantly at awakening administration, showing a greater sensitivity of SVR to physical activity. The *k*_*i*3_ parameter showed a lower dependence of SBP on HR in the administration at bedtime, that is, a lower baroreflex sensitivity than at awakening administration. On the other hand, a higher value of *k*_*i*4_ indicates a greater sensitivity of HR to changes in physical activity at bedtime and less at awakening administration. Ultimately, parameter *k*_2_ changed significantly at bedtime and not at awakening administration.

### 3.2 Estimated parameters for Ta-dependent effect model

**Figure 1:**
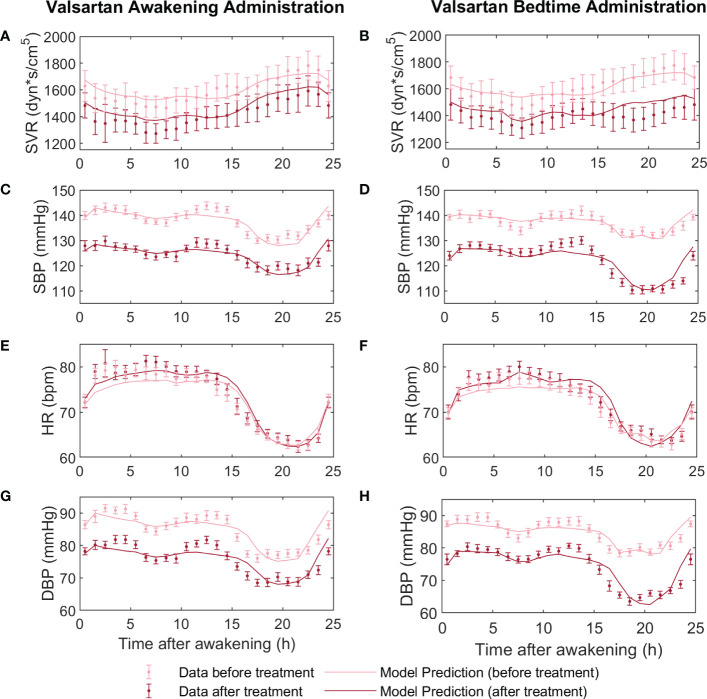
Fitting models to data before and after valsartan treatment at awakening (on the left) versus at bedtime (on the right). Before-treatment data from AM and PM administration groups were fitted independently, to a non-Ta dependent effect model. After-treatment data were fitted to the Ta-dependent effect model, both awakening and bedtime administration simultaneously. In panels **(A, B)** the dynamics of the experimental data (light red points for before treatment and dark red points for after treatment) and predictions of the models (light red line for before treatment and dark red line for after treatment) of systemic vascular resistance (SVR), **(C, D)** systolic blood pressure (SBP), **(E, F)** heart rate (HR), and **(G, H)** diastolic blood pressure (DBP) are displayed.

**Figure 2:**
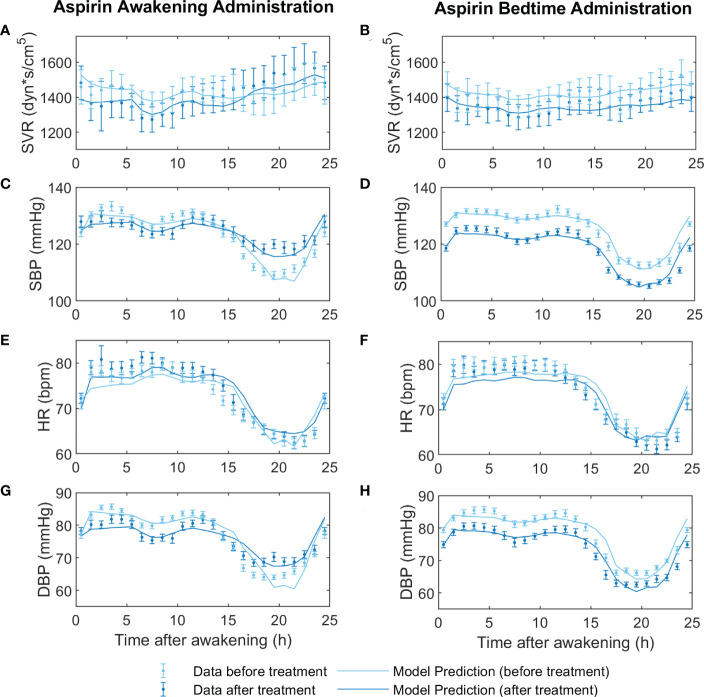
Fitting models to data before and after aspirin treatment at awakening (on the left) versus at bedtime (on the right). Before-treatment data from AM and PM administration groups were fitted independently, to a non-Ta dependent effect model. After-treatment data were fitted to the Ta-dependent effect model, both awakening and bedtime administration simultaneously. In panels **(A, B)** the dynamics of the experimental data (light red points for before treatment and dark red points for after treatment) and predictions of the models (light red line for before treatment and dark red line for after treatment) of systemic vascular resistance (SVR), **(C, D)** systolic blood pressure (SBP), **(E, F)** heart rate (HR), and **(G, H)** diastolic blood pressure (DBP) are displayed.

Since parameters *k*_*i*1_, *n, k*_*i*2_, *k*_*i*3_, *k*_2_ and *k*_*i*4_ changed significantly after treatment either at awakening or bedtime administration of aspirin/valsartan, a one-component circadian function for each of the parameters *k*_*i*1_, *n, k*_*i*2_, *k*_*i*3_, *k*_2_ and *k*_*i*4_ was included. **Table 3** shows the estimated parameters and respective CI for the circadian functions (equation 6) of the valsartan and aspirin models. **Figures 1**, **2** show the adjustment to data before treatment (non-Ta-dependent model) and after treatment (Ta-dependent model) for valsartan and aspirin, respectively. Solid lines represent model predictions and dots with error bars, the data with their respective standard errors.

**Table 3:** Estimated parameters of Ta-dependent effect model for valsartan and aspirin.

Parameter	Valsartan		Aspirin	
	Value	CI	Value	CI
A_n_	0.66	0.68	0.11	0.72
A_ki2_	1324.31	1181.65	535.47	1446.1
A_ki1_	259.94	242.67	751.10	548.74
A_ki3_	55.04	35.17	29.98	32.19
A_ki4_	5.97	2.17	7.69	2.20
A_k2_	1375.07	182.57	1111.28	182.01
O_n_	-105.36	0.63	-105.19	3.48
O_ki2_	-121.26	0.38	-119.43	3.37
O_ki1_	-135.96	0.89	-140.94	1.44
O_ki3_	-80.50	0.27	-77.38	0.44
O_ki4_	-193.31	0.22	-203.56	0.18
O_k2_	-77.44	0.05	-77.34	0.07

**Figure 3:**
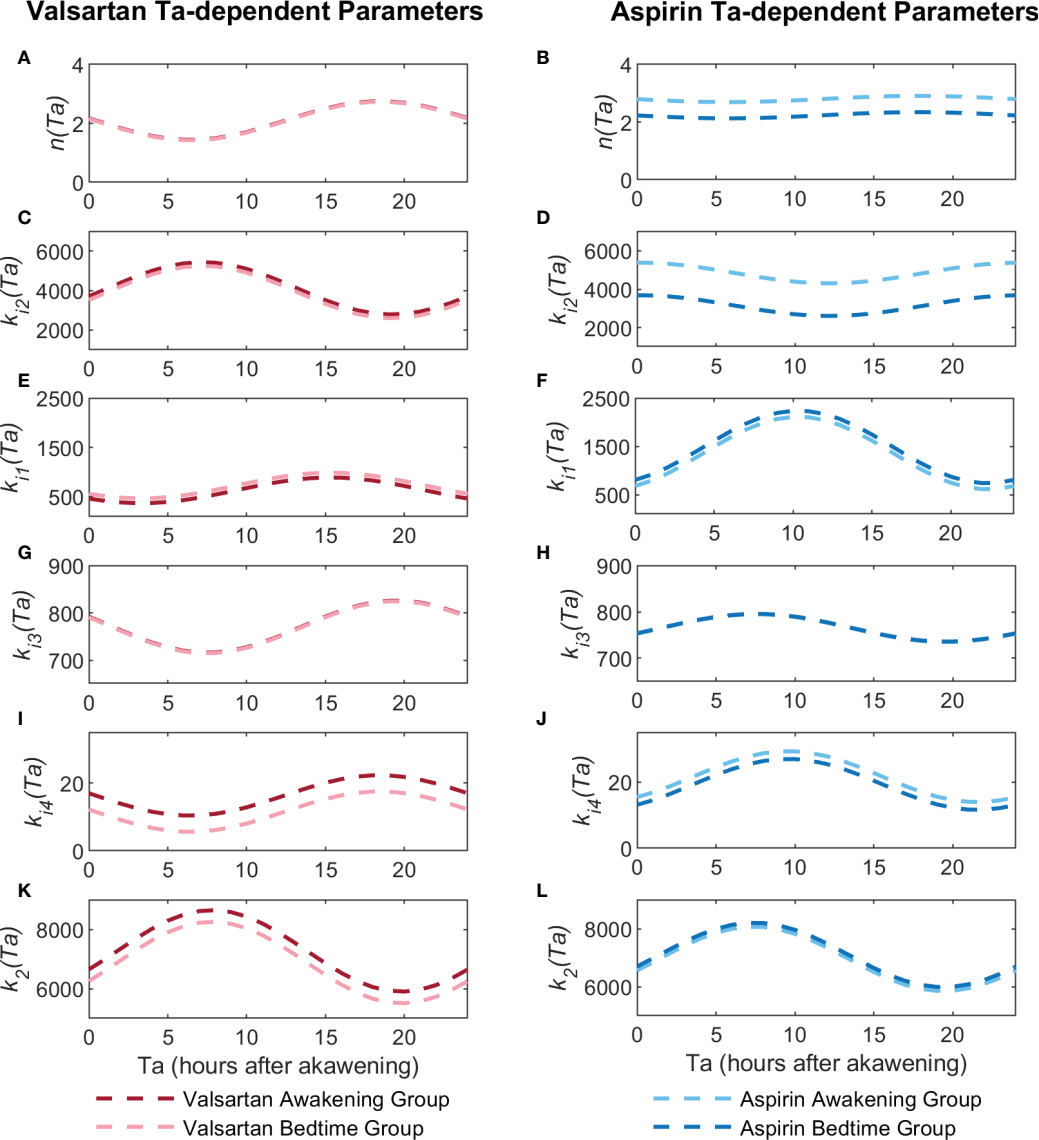
Ta-dependent parameter profiles (p(Ta)) for the valsartan and aspirin models. Figures **(A, C, E, G, I, K)** show the parameter profiles of the *n*, *k*_*i*2_, *k*_*i*1_, *k*_*i*3_, *k*_*i*4_ and *k*_2_ for valsartan, respectively. Figures **(B, D, F, H, J, L)** show the parameter profiles of the *n*, *k*_*i*2_, *k*_*i*1_, *k*_*i*3_, *k*_*i*4_ and *k*_2_ for aspirin, respectively.

Confidence intervals of estimated parameters showed that all but the parameter *A*_*n*_ were significantly different from 0 for the valsartan model, and all but *A*_*n*_, *A*_*ki*2_ and *A*_*ki*3_ for the aspirin model. The *p*(*Ta*) profiles for valsartan and aspirin parameters are shown in **Figure 3**. Higher values of *A* parameters indicate a greater dependence on the dosing time, while values of *O* parameters indicate the phase shift of the cosine function. Parameters *A*_*ki*2_, *A*_*ki*3_ and *A*_*k*2_ were higher for the valsartan model, while *A*_*ki*1_ and *A*_*ki*4_ were higher for the aspirin model. Similar values of *O*_*n*_, *O*_*ki*2_ and *O*_*k*2_ parameters were obtained for valsartan and aspirin models. However, *O*_*ki*1_, *O*_*ki*3_ and *O*_*ki*4_ parameters showed small differences between the valsartan and aspirin models, revealing significant differences in circadian parameters between aspirin and valsartan models.

### 3.3 Sensitivity and identifiability analysis

**Figure 4:**
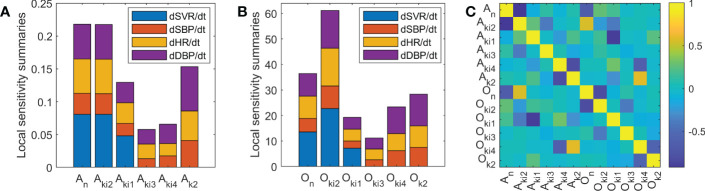
Sensitivity and identifiability analysis for Ta-dependent effect model of valsartan. **(A)** Local sensitivity summaries of *A* parameters. **(B)** Local sensitivity summaries of *O* parameters. **(C)** Correlation matrix between model parameters.

**Figure 5:**
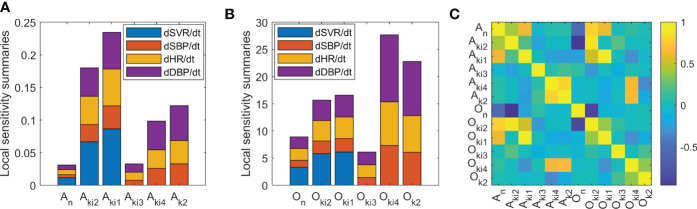
Sensitivity and identifiability analysis for Ta-dependent effect model of aspirin. **(A)** Local sensitivity summaries of *A* parameters. **(B)** Local sensitivity summaries of *O* parameters. **(C)** Correlation matrix between model parameters.

In order to identify parameters with higher circadian dependence, a local sensitivity analysis of Ta-dependent model parameters was performed. Summaries of the local sensitivity of parameters *A* and *O* are shown in **Figures 4A, B** for valsartan and **Figures 5A, B** for aspirin, respectively. Higher sensitivities for *O* parameters than for *A* parameters were obtained for both valsartan and aspirin Ta-dependent models. The parameters of the Ta-dependent model with higher sensitivity were *O*_*ki*2_ and *O*_*n*_ for valsartan, and *O*_*ki*4_ and *O*_*k*2_ for aspirin. Regarding identifiability analysis results, shown in **Figures 4C**, **5C**, the absolute values of correlations between parameters were less than 0.95 for the valsartan Ta-dependent effect model, and only the *A*_*ki*2_ and *O*_*n*_ parameters showed an absolute value greater than 0.95 (-0.96) for the aspirin Ta-dependent effect model.

### 3.4 Ta-dependent effect model simulations

**Figure 6:**
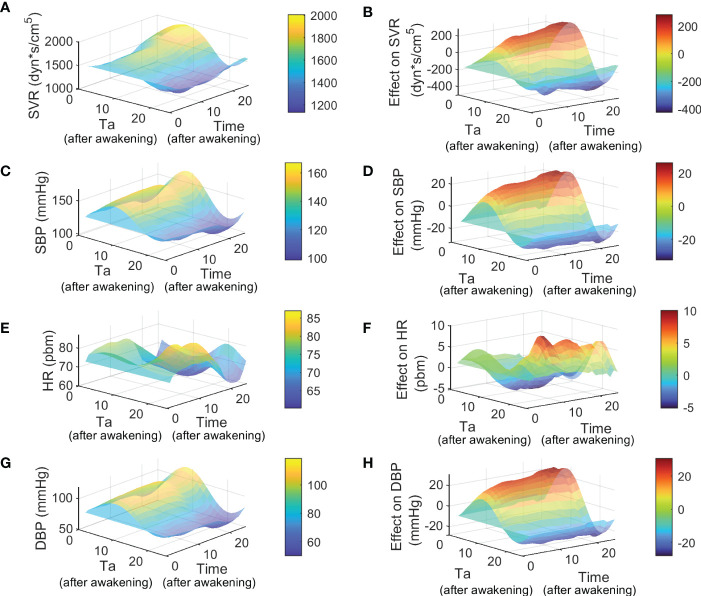
Simulation and effect results of Ta-dependent effect model of valsartan using *p*_*before*_ of awakening group. Figures **(A, C, E, G)** show the 24 hours simulation results (hours after awakening) of SVR, SBP, HR and DBP for different Ta from 0 to 24 hours (hours after awakening), respectively. Figures **(B, D, F H)** show the 24 hours effect results (hours after awakening) of SVR, SBP, HR and DBP for different Ta from 0 to 24 hours (hours after awakening), respectively.

**Figure 7:**
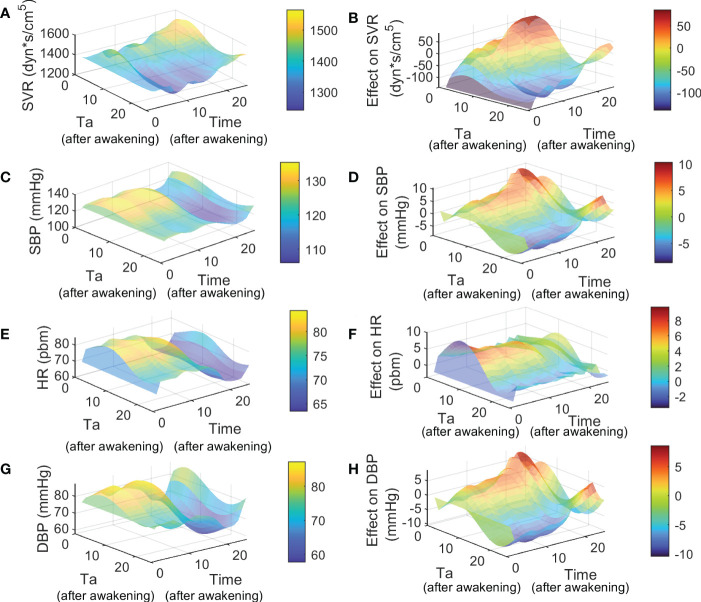
Simulation and effect results of Ta-dependent effect model of aspirin using *p*_*before*_ of awakening group. Figures **(A, C, E, G)** show the 24 hours simulation results (hours after awakening) of SVR, SBP, HR and DBP for different Ta from 0 to 24 hours (hours after awakening), respectively. Figures **(B, D, F, H)** show the 24 hours effect results (hours after awakening) of SVR, SBP, HR and DBP for different Ta from 0 to 24 hours (hours after awakening), respectively.

Using estimated parameters from **Table 3** and before-treatment parameters (*p*_*before*_) for Ta-dependent models of valsartan and aspirin, dynamics of SVR, SBP, HR, and DBP were obtained for the full range of Ta. Moreover, simulations of SVR, SBP, HR, and DBP before and after treatment allowed us to get the effect on the dynamics of these variables through time (equation 11). **Figures 6**, **7** show the simulation and effect results using *p*_*before*_ for awakening administration groups of aspirin and valsartan models and similar results using *p*_*before*_ for bedtime administration groups are attached in **Supplementary Figures 1**, **2** for valsartan and aspirin, respectively. Simulations of dynamics of SVR, SBP, HR, and DBP after treatment through the Ta range of 0 to 24 hours are shown in **Figures 6A, C, E, G** for valsartan and **Figures 7A, C, E, G** for aspirin. Predicted effect results are shown in **Figures 6B, D, F, H** for valsartan and **Figures 7B, D, F, H** for aspirin. Variations of DBP dynamics and effects of valsartan and aspirin models were found to be similar to those obtained for SBP across the entire Ta range.

On the one hand, the highest values of SVR, SBP, and DBP were obtained for valsartan Ta between 5 - 11 hours after awakening, and at the same time range, the lowest HR values were obtained. The lowest SVR, SBP, and DBP values (and higher effects) were predicted when valsartan is administered during the night rest (15-24 hours after awakening), at which time the highest HR values were also obtained. Because the administration of oral medications during night rest is infeasible, the predicted effects of bedtime and awakening administration were compared. The valsartan model predicted that bedtime administration has a slightly greater effect on SVR, SBP, and DBP by increasing the nocturnal dip and maintaining its effect during the morning rise of BP. On the other hand, lower values and greater effects of aspirin on SVR, SBP, HR, and DBP were observed between 15-24 hours after waking up, that is, between just before bedtime and before waking up. The highest values and minor effects on SVR, SBP, HR, and DBP were observed between 5 and 13 hours after awakening. Therefore, aspirin administration at bedtime showed better SVR, HR, and BP results. Notably, administration around 5-10 hours after awakening caused an increase in SVR and BP during the night rest for aspirin and valsartan treatments.

## 4 Discussion

Dosing-time optimization of antihypertensive medications has been proposed to reduce long-term cardiovascular risk by clinical and computational studies (5, 10–12). Some have shown that bedtime administration compared to awakening, improves cardiovascular risk in the long-term (2, 30), while others found no difference (31). The latter may be due to the different BP profiles of patients under study; i.e., a non-dipper patient probably will not show the same effect at a particular Ta compared to a dipper patient. Indeed, PK-PD models that include the circadian rhythm have shown that optimal Ta must be proposed in a personalized way (12). However, the causes and mechanisms involved in the dosing-time-dependent effect of antihypertensive medications have not been studied in depth. In this work, we propose a Ta-dependent model to analyze and predict the antihypertensive effect of valsartan and aspirin in subjects with grade I or II essential hypertension. The Ta-dependency was included in each model by using a periodic function for those parameters of the previously developed ODEs model that changed significantly after awakening or bedtime administration (*n*, *k*_*i*1_, *k*_*i*3_, *k*_2_ and *k*_*i*4_, see **Tables 1**, **2** **).** Periodic function parameters were estimated by fitting the Ta-dependent model to data of SVR, SBP, HR, and DBP after awakening and bedtime administration of valsartan and aspirin, obtaining good fitting results (**Figures 1**, **2**, and **Table 3**). However, differences in parameter values, significance, and phase shift of circadian functions were found between the valsartan and aspirin models.

Parameter *n* changed significantly in both awakening and bedtime administration groups but *A*_*n*_ was not significantly different from 0 for both valsartan and aspirin Ta-dependent models. Therefore, administering valsartan or aspirin changes the sensitivity of SVR to changes in glycemia (valsartan increases and aspirin decreases its sensitivity). However, this change is independent of dosing-time (Ta). Regarding *k*_*i*2_ parameter, it changed significantly for valsartan administration at awakening and bedtime and only at bedtime for aspirin. The *A*_*ki*2_ parameter was found to be significant only for the valsartan model. A higher value of *k*_*i*2_ (over the range of SVR values) implies a lower auto-regulatory effect of SVR. Therefore, at the peak of *k*_*i*2_(*Ta*) there is a lesser vasodilatory effect of valsartan. **Figure 3C** shows that the *k*_*i*2_(*Ta*) peak is approximately 7-8 hours after awakening, roughly the same time as the serum nitrite concentration peak occurs (32). Therefore, the lesser effect of valsartan when administered 7-8 hours after awakening could be explained by the fact that just at that moment, there is the maximum effect of nitric oxide on the vasculature. Furthermore, the antagonistic action of valsartan on the angiotensin type 1 receptor could reduce the peak of the vasoconstrictor effect of angiotensin II and norepinephrine that occurs in the morning when it is administrated at bedtime (33–36).

**Figures 3E, I** show the variation profiles of *k*_*i*1_(*Ta*) and *k*_*i*4_(*Ta*) when valsartan is administered at different dosing times. These profiles are similar between them and opposite to the *k*_*i*2_(*Ta*) profile. Since *k*_*i*1_ and *k*_*i*4_ are parameters that relate the sensitivity of SVR and HR to changes in physical activity, the greater vasodilation (lower *k*_*i*2_) the lesser impact of physical activity on SVR and HR (because of the same vasodilatory effect of exercise), that is, *k*_*i*1_ and *k*_*i*4_ parameters are greater and less sensitive.

On the other hand, for the aspirin model, Ta is significantly dependent on *k*_*i*1_ despite not being dependent on *k*_*i*2_. **Figures 3D, F, J** show a relationship between Ta dependency patterns, *k*_*i*2_(*Ta*) as a pattern opposite to *k*_*i*1_(*Ta*) and *k*_*i*4_(*Ta*). But in this case, the maximum of *k*_*i*2_(*Ta*) and the minimum of *k*_*i*1_(*Ta*) and *k*_*i*4_(*Ta*) occur upon awakening. Therefore, higher values of *k*_*i*2_ and lower values of *k*_*i*1_ and *k*_*i*4_, that is less effect on SVR and HR, occurs when aspirin is administered upon awakening. The circadian rhythm of *k*_*i*2_ in the aspirin model could be related to the coincident circadian rhythm of the total tissue factor pathway inhibitor (a coagulation inhibitor), morning hypercoagulability and also to the peak production and release of platelets during the night rest (32, 37, 38). Due to the rapid absorption and elimination of aspirin, morning administration would not allow the inhibition of cyclooxygenase 1 and the production of thromboxane A2 by the new platelets produced during the night and in the early morning, decreasing their anticoagulant effect (37). Indeed, Bonten et al. demonstrated a significantly greater anticoagulant effect during morning hours when aspirin is administered at bedtime (39). Therefore, a better anticoagulant effect when aspirin is administrated at bedtime would produce a lower blood viscosity, affecting hemodynamic responses of SVR, BP and HR to physical activity, but without significantly affecting the regulation of vasodilation. However, the results from different studies are contradictory; some report an antihypertensive effect of aspirin when administered at bedtime and some report the absence of it (8, 39). The antihypertensive effect of aspirin could be explained by differences between the studied population and the subjects’ physical activity (6–8, 39, 40). On the other hand, it has been stated that clinical trials that suggest an antihypertensive effect of aspirin are uncontrolled, unmasked, and potentially biased (41). Thus, biased clinical study results could lead to erroneous inferences about the antihypertensive effect and its relationship with the circadian rhythm. Therefore, it is encouraged to carry out this Ta-dependent effect analysis on reliable clinical trial data.

Parameters related to baroreflex sensitivity *k*_*i*3_ and baseline HR *k*_2_ are shown in **Figures 3G, K** for valsartan and **Figures 3H, L** for aspirin. Parameters *k*_*i*3_ and *k*_2_ had significant changes in both awakening and bedtime valsartan administration, and both *A*_*ki*3_ and *A*_*k*2_ were significantly different from 0. Therefore, valsartan administration improves baroreflex sensitivity in a Ta-dependent manner, probably because valsartan inhibits the positive action of angiotensin receptor type 1 on NE release from sympathetic nervous system neurons (34). In addition, lower *k*_2_ values are obtained when valsartan is administered overnight, with the aim of compensating for changes in *k*_*i*3_ and BP. In the case of aspirin, significant changes in parameters *k*_*i*3_ and *k*_2_ were found after administration upon awakening and at bedtime. However, only the parameter *A*_*k*2_ was significantly different from 0, so aspirin only improves baroreflex sensitivity when administered upon awakening. This increased sensitivity may be linked to reduced thromboxane A2 production, which participates in metabolite sensitization of muscle mechanoreceptors (42).

In accordance with the main mechanisms of action related to each drug studied, the higher sensitivity of *k*_*i*2_ and *k*_*i*4_ were found for valsartan and aspirin, respectively (**Figures 4**, **5**). The identifiability analysis results showed that all correlation values were less than 0.95, except for the correlation between the parameters *A*_*ki*2_ and *O*_*n*_ from the aspirin model (-0.96). Hence, the Ta-dependent model has no structural identifiability problems. Subsequently, Ta-dependent models for valsartan and aspirin allowed us to predict and analyze the impact of Ta on SVR, BP, and HR dynamics (**Figures 6**, **7**). Higher effects on SVR, SBP, and DBP were predicted when valsartan or aspirin are administered at bedtime. However, increased values of SVR and BP during the night rest were observed for dosing times 5-10 hours after awakening, both for aspirin and valsartan treatments. Compensatory regulation mechanisms and the absence or lower plasma concentration of the drug during the night could explain the increase in SVR and nocturnal BP for dosing times of 5-11 hours. However, further studies are necessary to clarify the phenomenon and the mechanisms involved. Finally, better results in bedtime administration of valsartan and aspirin to patients with grade I or II essential hypertension were found in this study (non-dippers for valsartan and dippers for aspirin). Still, they are due to different mechanisms of action and dependence on circadian phases. Therefore, drugs with different mechanisms of action and pharmacokinetics could present different Ta-dependence and, therefore, different optimal administration times.

## 5 Conclusion

In summary, this work presents a mathematical model able to simulate the Ta-dependent effect of antihypertensive drugs. In addition, since the developed model is based on physiological BP regulation mechanisms, the values and sensitivity of the parameters allow us to infer possible circadian regulation mechanisms that affect the BP therapeutic outcome. The adjustment and simulation of the model developed to the data of each patient allows not only prediction of the BP profiles but also finds a theoretical optimal administration time that could be tested in future clinical trials.

## Data availability statement

Publicly available datasets were analyzed in this study. This data can be found here: https://www.synapse.org/#!Synapse:syn46580597/files/.

## Author contributions

All authors listed have made a substantial, direct, and intellectual contribution to the work and approved it for publication.
