# Supplementary material for: Understanding the dosing-time-dependent antihypertensive effect of valsartan and aspirin through mathematical modeling
Source: Front Endocrinol (Lausanne). 2023 Mar 8;14:1110459. doi: 10.3389/fendo.2023.1110459 (PMC10031009; doi:10.3389/fendo.2023.1110459)
Supplement: Supplementary file 1 [file DataSheet_1.docx]

Supplementary Material

Understanding the dosing time-dependent antihypertensive effect of valsartan and aspirin through mathematical modeling

**Cortés-Ríos, J. and Rodriguez-Fernandez, M.^*^**

*** Correspondence:** Corresponding Author: [marodriguezf@uc.cl](mailto:marodriguezf@uc.cl)


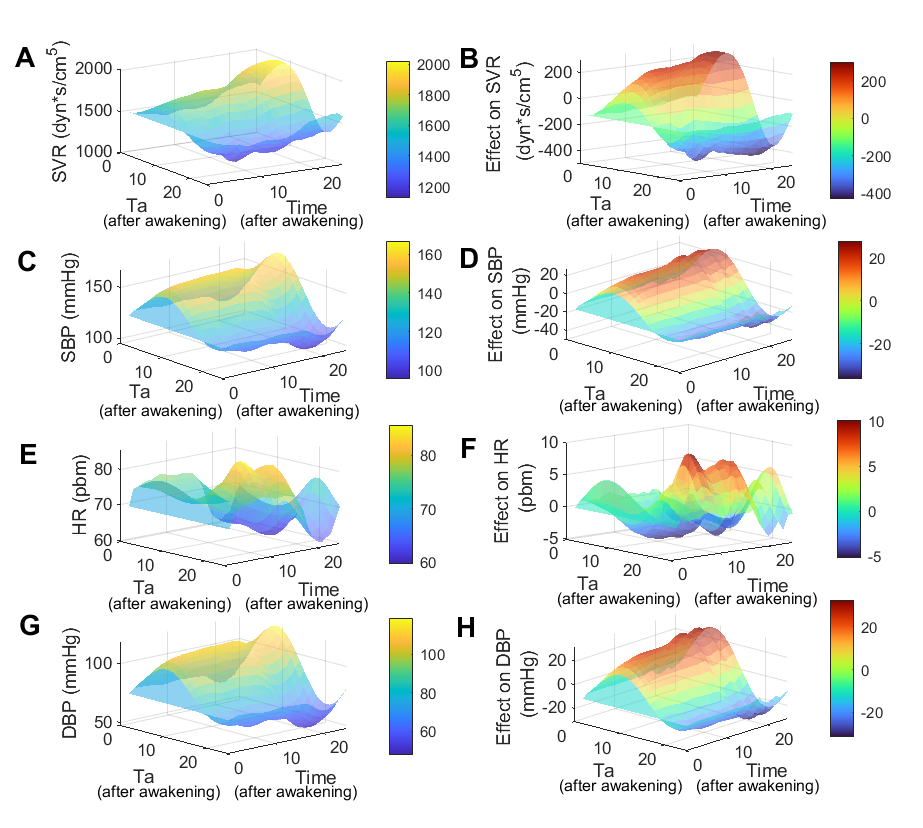


**Supplementary Figure 1.** Simulation and effect results of Ta-dependent effect model of valsartan using $p_{before}$ of bedtime group. Figures **(A)**, **(C)**, **(E)** and **(G)** show the 24 hours simulation results (hours after awakening) of SVR, SBP, HR and DBP for different Ta from 0 to 24 hours (hours after awakening), respectively. Figures **(B)**, **(D)**, **(F)** and **(H)** show the 24 hours effect results (hours after awakening) of SVR, SBP, HR and DBP for different Ta from 0 to 24 hours (hours after awakening), respectively.

**
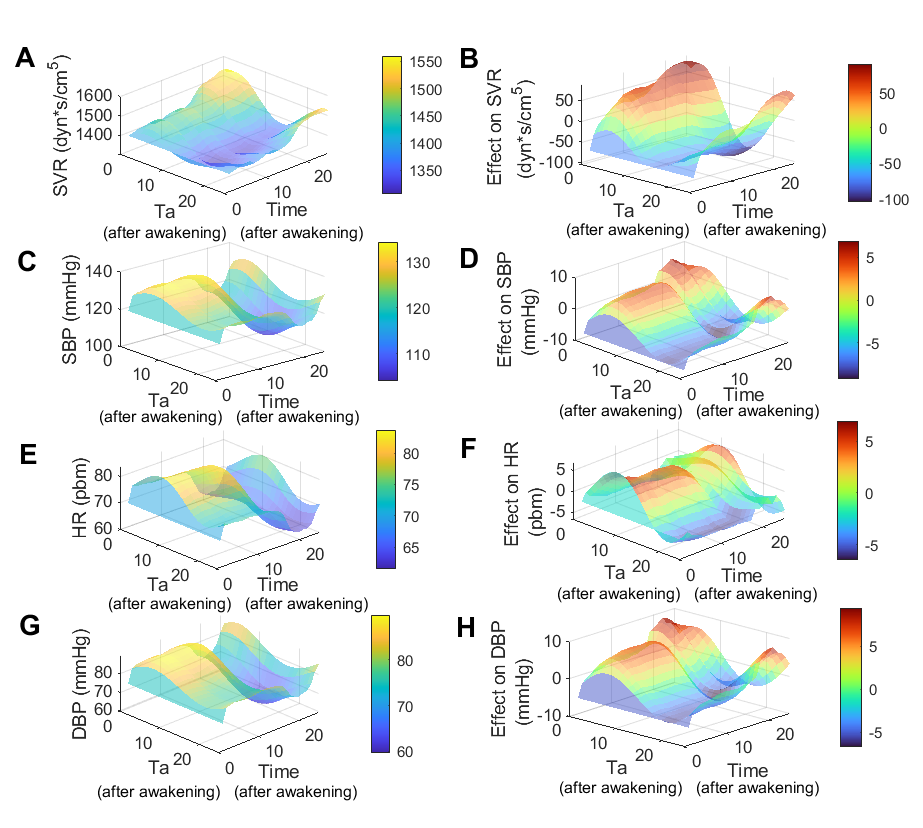
**

**Supplementary Figure 2.** Simulation and effect results of Ta-dependent effect model of aspirin using $p_{before}$ of bedtime group. Figures **(A)**, **(C)**, **(E)** and **(G)** show the 24 hours simulation results (hours after awakening) of SVR, SBP, HR and DBP for different Ta from 0 to 24 hours (hours after awakening), respectively. Figures **(B)**, **(D)**, **(F)** and **(H)** show the 24 hours effect results (hours after awakening) of SVR, SBP, HR and DBP for different Ta from 0 to 24 hours (hours after awakening), respectively.
